# Typhoid Infection in a Two and a Half Year Old Child from Drinking Milk Not Thoroughly Boiled

**Published:** 1896-12

**Authors:** 


### ABSTRACTS FROM FOREIGN JOURNALS.

UNDER THE DIRECTION OF
J. PRESTON HOSKINS, PRINCETON, N. J.

TYPHOID INFECTION IN A TWO AND A HALF YEAR OLD CHILD FROM DRINKING MILK NOT THOROUGHLY BOILED. In May, 1892, a girl two and a half years old became suddenly sick with symptoms of irritation of the bowels, relieved by evacuations and emissions of urine. The treatment adopted, in which other food replaced milk, quickly diminished the symptoms, which reappeared again as soon as milk was given. Marked typhoid infection also developed, lasting for some weeks. At the end of the fifth week the child was convalescent. For the reason that the child had been fed exclusively with milk, and because the symptoms after disappearing suddenly reappeared when the milk-diet was recommenced, the physician deemed himself justified in concluding that the milk contained the injurious elements. A further reason was found in the fact that the four-year-old sister of the patient, who had drunk of the same milk, but not such a large quantity, also suffered from diarrhœa at the same time that her younger sister was sick. The bacteriological investigation of the suspected milk and evacuations of the patient gave the following results: In the milk a bacterium was found in great quantity which presented the morphological and biological characteristics of the bacterium coli commune. In the evacuations the same bacteria were found, as well as in the blood of mice which had been infected with the fecal matter of the patient. It is a noteworthy fact that these bacteria have been found in a virulent condition in milk which had been boiled. This can only be explained on the ground that the milk had not been boiled enough, or that the boiled milk after it had become cool had been poured back into the can, which had not been cleansed sufficiently.—REHN *in Veeartsenijkundige Bladen voor Nederlandsch-indie*, Deel ix., aflevering 4.

